# Protein Oxidation in Aging and Alzheimer’s Disease Brain

**DOI:** 10.3390/antiox13050574

**Published:** 2024-05-07

**Authors:** Rukhsana Sultana, D. Allan Butterfield

**Affiliations:** 1 Department of Neuroscience, School of Behavioral and Brain Sciences, The University of Texas at Dallas, 800 West Campbell Rd., Richardson, TX 75080, USA; rukhsana.sultana@utdallas.edu; 2 Department of Chemistry, and Sanders-Brown Center on Aging, University of Kentucky, Lexington, KY 40506, USA

**Keywords:** protein oxidation, protein carbonylation, protein nitration, Alzheimer’s disease, mild cognitive impairment, reactive nitrogen species, reactive oxygen species, amyloid β-peptide, aging

## Abstract

Proteins are essential molecules that play crucial roles in maintaining cellular homeostasis and carrying out biological functions such as catalyzing biochemical reactions, structural proteins, immune response, etc. However, proteins also are highly susceptible to damage by reactive oxygen species (ROS) and reactive nitrogen species (RNS). In this review, we summarize the role of protein oxidation in normal aging and Alzheimer’s disease (AD). The major emphasis of this review article is on the carbonylation and nitration of proteins in AD and mild cognitive impairment (MCI). The oxidatively modified proteins showed a strong correlation with the reported changes in brain structure, carbohydrate metabolism, synaptic transmission, cellular energetics, etc., of both MCI and AD brains compared to the controls. Some proteins were found to be common targets of oxidation and were observed during the early stages of AD, suggesting that those changes might be critical in the onset of symptoms and/or formation of the pathological hallmarks of AD. Further studies are required to fully elucidate the role of protein oxidation and nitration in the progression and pathogenesis of AD.

## 1. Introduction

Living cells are composed of biomolecules, i.e., proteins, lipids, carbohydrates, and nucleic acids. Each biomolecule has a defined role in regulating the normal cellular and physiological functions. Proteins are essential molecules that play crucial roles for maintaining cellular homeostasis and carrying out biological functions such as catalyzing biochemical reactions, structural proteins, immune response, etc. However, proteins are highly susceptible to damage through a process called protein oxidation. In a normal cell, there is normally a balance between the amount of the reactive oxygen species (ROS)/reactive nitrogen species (RNS) produced and the antioxidant defense systems. But, under certain conditions, this balance between ROS and RNS is lost, resulting in oxidative stress or nitrosative stress.

**Figure 1:**
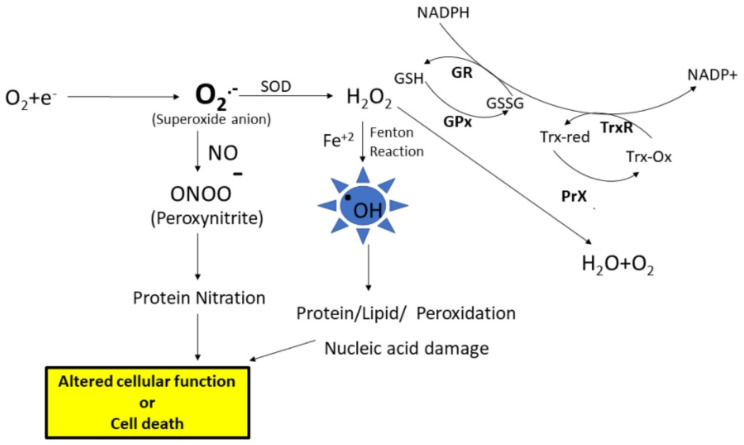
The electron transport chain in the mitochondria is the main source for the formation of superoxide anion radicals. The superoxide anion is converted to hydrogen peroxide (H_2_O_2_) by the superoxidase dismutase (SOD) enzyme. Hydrogen peroxide is converted to water and oxygen by the glutathione peroxidase (GPx) enzyme or peroxiredoxin (PrX). Superoxides can react with nitric oxide, forming peroxynitrite, which leads to that nitration of proteins. Further, high levels of H_2_O_2_ lead to increased formation of hydroxyl radicals (^.^OH), which leads to biomolecule modifications, subsequent alteration of cell function, or cell death. GSH—Reduced glutathione, GSSG—oxidized glutathione, GR—glutathione reductase, TrxR—thioredoxin reductase.

There are many sources for the production of free radicals; one of the major sources is the leakage of superoxide radical from the mitochondrial electron transport chain. Under physiological conditions, superoxide anion radical (O_2_^.−^) levels are maintained in the cell by the antioxidant enzyme, superoxide dismutase (SOD), which disproportionates O_2_^.−^ to hydrogen peroxide (H_2_O_2_) and oxygen (Figure 1). Further, the formed H_2_O_2_ is converted to water and oxygen by the catalase or peroxidase enzymes. Another enzyme that converts H_2_O_2_ to water is glutathione peroxidase (GPx), which uses reduced glutathione (GSH). Further, the GSH level is maintained by the enzyme glutathione reductase (GR), which converts oxidized glutathione (GSSG) to GSH using NADPH for reducing equivalents. However, as stated earlier, an imbalance leads to increased production of free radicals, eventually leading to ROS and RNS. ROS such as superoxide anion radicals can interact with iron–sulfur clusters, resulting in the release of free iron [1] (Figure 1). When redox divalent metal ions like Fe^+2^ or Cu^+2^ are available in the nearby vicinity of hydrogen peroxide, the Fenton reaction is engaged, resulting in the formation of highly damaging hydroxyl radicals. These hydroxyl radicals can react with biomolecules including proteins, lipids, carbohydrates, and nucleic acids, leading to functional impairment of these biomolecules [2]. Further, if the levels of superoxide radicals are high, and if there is an increased availability of nitric oxide, radical–radical recombination results in the formation of peroxynitrite, a highly reactive product with a half-life of less than 1s that can lead to protein or lipid nitration. The end products of oxidative and nitrosative stress, along with altered levels of antioxidant enzymes and elevation of cellular stress response proteins, serve as good indicators of damage to cells and their subsequent fate.

Increased ROS can lead to peptide backbone fragmentation, hydrogen atom abstraction at alpha carbons, and attack on several amino acid side chains (Lys, Arg, Pro, Thr, etc.), resulting in the formation of protein carbonyls. Additionally, protein carbonyls can also be formed via the Michael adducts formation between Lys, His, or Cys residues and α- and β-unsaturated aldehydes formed during the peroxidation of polyunsaturated fatty acids [3,4,5,6]. Further, protein carbonyls are also formed via the secondary reactions of amino groups of lysine residues with reducing sugars or their oxidation production (glycation/glycoxidation reactions) [7,8,9,10]. Most oxidative protein damage is irreversible; however, there are certain enzymes in vivo that can either repair or clear the oxidatively damaged proteins. Protein carbonyls are stable products of protein oxidation compared to the other products of oxidative stress, e.g., F2 isoprostanes, which are readily generated during sample storage, processing, and analysis. Consequently, protein carbonyls are a general and widely used index to determine the extent of oxidative modification both under in vivo and in vitro conditions. Several sensitive assays were developed for the detection of oxidatively modified proteins. Protein carbonyls are most often detected by derivatization of the carbonyl group with 2,4-dinitrophenylhydrazine (DNPH), which leads to the formation of protein hydrazones [11]. These protein hydrazones can be detected spectrophotometrically at 375 nm, but in solution samples, homogeneity or uniformity is one of the main issues for spectroscopic analysis. Another means for the detection of protein hydrazones is immunochemical detection using an anti-DNP-protein antibody that can provide a clear indication of the amount of total protein carbonyls in a given sample [12]. The latter method has been widely employed to detect protein carbonyls in biological samples. Other methods for the analysis of protein carbonyls include the use of biotin hydrazide coupled to FITC-labelled streptavidin [13].

Nitric oxide is multifunctional; it is involved in signal transduction by activating guanylate cyclase and increasing intracellular cGMP. NO at normal physiological levels is neuroprotective and plays an important role in vasodilation, neurotransmission, cardiac function, and inflammation; however, as noted above, NO, at excess concentrations, reacts with superoxide to form the highly reactive peroxynitrite, contributing to the oxidative stress [14,15,16]. Nitric oxide is produced from arginine in a reaction catalyzed by the enzyme nitric oxide synthase (NOS), of which there are three different types: endothelial and neuronal NOS (eNOS and n-NOS, respectively, constitutively produce NO) and inducible by iNOS. NO has been associated with neurodegenerative diseases by acting as a neurotoxin when produced excessively; however, other studies suggest that NO may have neuroprotective properties as well [17,18,19]. Peroxynitrite can cause oxidation of amino acids such as cysteine and methionine, as well as nitration of tyrosine and tryptophan. The presence of 3-nitrotyrosine in proteins can lead to altered protein structure and function. The nitration of tyrosine residues can disrupt protein–protein interactions and enzymatic activities. Additionally, 3-nitrotyrosine-facilitated steric hinderance modifications can impact signaling pathways mediated by tyrosine phosphorylation, affecting cellular communication and regulation of other cellular functions such as microtubule assembly and ATPases. 3-NT is a marker of oxidative damage in biological systems.

Protein oxidation (indexed by protein carbonyls and 3-NT) can have detrimental effects on protein structure and function. As mentioned earlier, proteins are important in regulating vital cellular processes such as structure, signal transduction, and metabolic processes. In the case of the nervous system, proteins are critical for synaptic communication, transportation of molecules, etc. When proteins are carbonylated, the overall polarity of the protein is affected, which may lead to alterations in the secondary, tertiary, and quaternary structure of the proteins. For example, when additional dipole moments are introduced into a protein due to oxidative modification, buried hydrophobic amino acid residues will be exposed to the aqueous environment, which may promote protein aggregation and accumulation of the oxidized proteins as cytoplasmic inclusions. Further, the altered structure of oxidized proteins may lead to decreased protein degradation via proteasome or autophagy pathways, leading to increased accumulation of the oxidized protein within neurons, further contributing to the cellular dysfunction.

The oxidation of proteins has previously been shown to lead to alterations in protein expression and gene regulation, protein turnover, modulation of cell signaling, etc., eventually leading to compromised cellular function and cell death. These oxidative modifications can alter protein stability, activity, and interactions with other molecules. Oxidized proteins can also trigger an inflammatory response in the brain, activating microglia and the release of pro-inflammatory cytokines [20]. Chronic neuroinflammation associated with protein oxidation can exacerbate neuronal damage and contribute to the progression of neurodegenerative disorders [21]. As a result, protein oxidation can contribute to the development of various diseases, including neurodegenerative disorders, cardiovascular diseases, and cancer [22].

## 2. Protein Oxidation in Aging Brain

The aging brain is more sensitive to oxidative damage, particularly neurons that are non-dividing, post mitotic cells [23]. Progressive impairment of mitochondrial function has been implicated in aging and age-related disorders [24,25]. Mitochondria are a major internal source for ROS and hence are also a major target of oxidative damage. ROS also plays an important role in cell signaling. However, during aging and under increased environmental stress, ROS levels can increase dramatically, leading to significant damage to biomolecules. Analysis of mitochondria from different brain regions of young, middle-aged, and old rats revealed that mitochondria from the cerebral cortex of the old rats produce more ROS and exhibit mitochondrial swelling due to increased calcium load when compared to younger rats [26]. Further, mitochondria located in the presynaptic terminals are exposed to high levels of calcium due to the synaptic transmission process, related to the opening of voltage-gated calcium channels, which can further accelerate the processes of neuronal oxidative damage and eventual cell loss by activating the calcium-dependent proteases [23]. In addition to self-damage, mitochondrial-generated ROS can damage nearby biomolecules, thereby amplifying the effects free radicals exert on the overall performance of the cell.

Protein oxidation has been associated with age-dependent cognitive decline. Studies showed that oxidative modifications, such as protein carbonylation and 3-nitrotyrosine formation, accumulate in aging tissues, including the brain [5,27]. Protein carbonylation occurs as a prominent oxidative modification as a result of elevated ROS in the aging brain, which is further supported by a decline in antioxidant systems such as superoxide dismutase and catalase [3,4]. The levels of NO gradually decrease with increasing aging in the hippocampus and cortex [28]. Oxidative damage to key proteins due to increasing age is involved in learning and memory processes and can impair synaptic plasticity and neuronal communication, leading to deficits in cognitive function [3]. Aging neurons are particularly susceptible to the effects of protein oxidation due to a decline in antioxidant defenses and repair mechanisms. Accumulated oxidative damage to proteins can accelerate the aging process of neurons and increase the risk of neurodegenerative diseases, such as Alzheimer disease, Parkinson disease, and amyotrophic lateral sclerosis [29]. Older mice show elevated levels of oxidative damage in tissues like the brain, liver, and heart, impacting their overall health and longevity [27]. Further, aged Norway rats also demonstrate heightened oxidative stress as they grow older, with age-associated declines in antioxidant defenses contributing to increased ROS levels [30,31]. Rats may exhibit oxidative damage in tissues involved in age-related diseases such as neurodegeneration and cardiovascular dysfunction. The aging neuron shows impaired glucose uptake, which may further compromise its ability to maintain ion homeostasis and other energy-related cellular processes [4]. The perturbed glucose metabolism may contribute to hyperphosphorylation of the microtubule-associated protein tau, leading to aggregation of the tau protein.

Senescence-accelerated mouse prone 8 (SAMP8) shows age-related cognitive decline and exhibits deficiency in learning and memory and has been used as a model for gerontological and age-related dementia research [32]. The age-associated changes in SAMP8 mice brains correlated with increased oxidative stress [33,34], which started as early as 4 months. SAMP8 mice showed an age-related increase in APP and Aβ peptide expression in the hippocampus; by 6 months of age, SAMP8 mice showed Aβ deposits as granules in the hippocampus, which formed plaques by 20 months [35,36,37,38]. The cognitive deficits that occur in this model were reported to occur before plaque formation, suggesting that soluble oligomers of Aβ induce oxidative damage, not the plaque, and are associated with learning and memory impairments. Consequently, the SAMP8 mouse is considered a model of oxidative stress and AD. Antisense oligonucleotide (AO) directed against the Aβ region of the APP gene in aged SAMP8 mice significantly decreased APP protein levels, reversed learning and memory deficits [36,38], and significantly decreased brain Aβ levels and oxidative stress biomarkers in age-matched SAMP8 mice that received a random AO [39]. Further, another study by Kumar et al. (2009) showed that the age-related increase in APP and Aβ may be due to an age-related increase in PS-1 protein levels in the hippocampus of SAMP8 mice [40]. These authors showed that AO directed against the PS-1 gene in 12-month-old SAMP8 mice resulted in significantly decreased PS-1 protein levels, oxidative stress parameters, and reversed learning and memory deficits in the hippocampus. Further, redox proteomics approaches identified lactate dehydrogenase 2 (LDH-2), creatine kinase (CK), DRP-2 (CRMP-2), and α-spectrin 2 as selective targets of protein oxidation (Carbonyls) in the brain of 12-month old SAMP8 mice compared to 4-month-old SAMP8 mice [39]. Further, the use of antisense oligonucleotide directed against the Aβ region of the APP gene in 12-month-old SAMP8 mice led to decreased carbonylation of aldolase 3 (Aldo 3), coronin 1a (Coro 1a), and peroxiredoxin 2 (Prx2) [41]. Additionally the nitration of LDH, DRP-1 (CRMP-1), dynamin-1 (Dyn-1), GFAP, vesicle-fusing ATPase (NSF), α-enolase, isocitrate dehydrogenase (IDH), actin-related protein 2 (Actr2), and septin-11 (SEP11) was significantly decreased in SAMP8 mice receiving AO directed against the PS-1 gene, while nitration of DRP-2 (CRMP-2) and peptidase α (DPL2) was significantly increased [42].

In summary, animal models of aging have shown that age plays an important role in elevating oxidative stress in biological cells, thereby contributing to neuronal dysfunction, cognitive decline, and the development of neurodegenerative diseases. Understanding the mechanisms of protein oxidation in the brain and exploring strategies to counteract its effects are crucial for promoting healthy brain aging and potentially delaying the onset of age-related neurological disorders.

## 3. Protein Oxidation in AD

Alzheimer’s disease (AD) is an age-related disease and the most common form of dementia in the elderly population. In the United States, over six million people are living with AD, and it is expected that the number of AD patients will increase to 13 million by 2050, unless means to delay the onset or progression of the disease are developed [42]. As AD progresses, patients present memory loss, cognitive impairment, aphasia, and changes in behavior that are not due to any other cause. Changes in the brain structure are observed using structural MRI studies, which clearly demonstrate early brain atrophy predominant in the hippocampus, precuneus, temporal and parietal lobes, and parts of the frontal cortex and cingulate gyrus. Interestingly, the cerebellum typically displays no or minor changes in AD brains compared to age-matched controls [43]. Further, FDG-PET analysis showed decreased glucose metabolism in parietal–temporal association cortices [44] in AD compared to control brain. Based on the structural and functional alterations assessed by MRI imaging and clinical signs and symptoms, individuals can be diagnosed with AD. A definitive diagnosis of AD is obtained at autopsy by histopathological presence of three main hallmarks of AD, i.e., extracellular senile plaques (SP, rich in amyloid β-peptide [Aβ])) and intracellular neurofibrillary tangles (NFTs, aggregate of hyperphosphorylated tau) and synapse loss [45]. Another way of confirming the diagnosis of AD involves analysis of CSF or plasma biomarkers [46,47].

Based on cognitive, structural, and histopathological testing, an arguably early stage of AD is identified, i.e., mild cognitive impairment (MCI). MCI is divided into two categories: amnestic MCI (aMCI) and MCI. Patients who have developed aMCI have an increased probability of developing clinical AD [48], yet it remains a challenge to identify which patients with MCI will proceed to clinical AD and which patients will not [49]. Individuals with MCI have decreased hippocampal volume [50], decreased energy metabolism, increased number of NFTs and subsequent Braak staging (Stage II-III) [51], lowered MMSE scores, and increased levels of amyloid deposition correlated with both non-amnestic MCI as well as amnestic MCI [52]. In addition to neuronal loss in AD and MCI brains, studies also reported degraded white matter in the prefrontal and parietal cortex, which led to an impaired ability to carry out independent financial management. This loss of white matter is a consequence of impaired function of neurons or a trigger to onset of AD and requires further study [53].

Even though much is known about the impact of AD on cognitive function, the exact molecular mechanisms that lead to the structural changes (loss of neurons, altered white matter) and development of AD pathology largely remain unknown. A number of mutations in genes including *presenilin-1* (PS-1), *presenilin-2* (PS-2), and *APP* genes cause familial AD (FAD) [54,55,56]. In addition, other genes linked to increased risk of AD include *apolipoprotein E* allele 4 (*APOE 4*), *phosphatidylinositol-binding clathrin assembly protein* (PICALM), *endothelial nitric oxide synthase-3*, *alpha-2-macroglobulin*, and *clusterin* (CLU aka APOJ), among others [57,58,59,60]. A number of hypothesized factors including amyloid β (Aβ), hyperphosphorylated tau, cholinergic neuron damage and oxidative stress, inflammation, etc., were developed to explain AD pathological lesions and clinical symptoms. All of these hypotheses are to some extent based on the role of Aβ [3].

**Figure 2:**
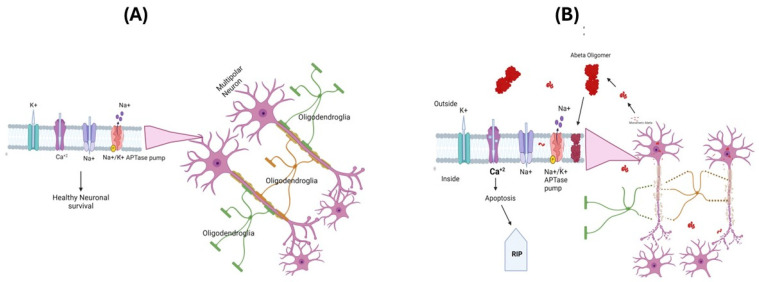
The neuronal membrane consists of a lipid bilayer with ion channels and protein transporters embedded in it. These play an important role in creating and maintaining membrane potentials and regulating other cellular functions (**A**). In Alzheimer’s disease, the oligomeric Aβpeptide inserts itself into the lipid bilayer, forming an ion channel that allows the free movement of ions across the membrane, altering the membrane potentials and promoting apoptosis and loss of neuronal cells (**B**). APP-Amyloid precursor protein.

The oxidative stress hypothesis for the pathogenesis and progression of AD [3,61,62] is based on the observations of increased cellular free radical production in AD brains and the identification of markers of oxidative and nitrosative stress, i.e., protein oxidation, lipid peroxidation, and nucleic acid oxidation [3,4,5,63]. In AD, loss of pyramidal neurons in the hippocampus, layer II entorhinal neurons, and large neurons in the temporal and frontal lobe association cortex were proposed to cause an imbalance in the amount of ROS production and antioxidant enzymes, leading to selective increased neural vulnerability, leading to a loss of cells [64,65]. Aβ(1-42) has been suggested as a key player in the oxidative stress observed in AD brains, and this is extensively supported through various studies of AD models [66,67,68,69,70,71]. The amyloid-β (Aβ) peptides Aβ(1-40) and Aβ(1-42) are major constituents of SP [72], since they are derived from APP and exhibit a tendency to aggregate and form monomers, oligomers, protofibrils, and fibrils. Recent data suggest that among these different states of aggregates, oligomeric Aβ is the most toxic species [67,73,74]. The oligomeric form of Aβ can insert itself into the cell membrane, altering the functions of various ion channels including Na^+^-K^+^ ATPase pump function, which can affect the resting membrane potential and ability of neuronal cells to fire and action potential [75]. Further functional alterations in ion channels like NMDA can lead to increased calcium accumulation in AD brains, triggering the onset of the cell death process via apoptosis [76,77] (Figure 2A,B).

The role of Aβ(1-42) peptide small oligomers in oxidative stress involves the single methionine residue at position 35 (Met-35) of the Aβ peptide^3^ [78,79,80]. An in vitro study showed that when Met at position 35 is substituted for norleucine, primary hippocampal cell culture showed no protein oxidation, neurotoxicity, or free radical formation, supporting the importance of Met-35 in inducing oxidative stress [78,80]. Additionally, an in vivo study of transgenic *Caenorhabditis elegans* carrying a human Aβ(1-42M35Cysteine) substitution showed lower levels of protein carbonyls in comparison to Aβ(1-42) transgenic animals [80,81]. Further, in vivo studies of a PDAPP transgenic mouse model of AD carrying the Swedish (670/671_KM→NL_) and Indiana (717_V→I_) familial forms of AD (APP_Sw,In_ /J20 Tg) demonstrated significant in vivo oxidative damage [82]. In constrast, when a third mutation to *APP* in PDAPP mice was introduced in which M35 was substituted by Leucine [Aβ(M35L], corresponding to residue 631 of human *APP*), in vivo brain oxidative stress was abolished [83]. One possible mechanism that was proposed is the ability of small oligomers of the relatively hydrophobic Aβ(1-42) to insert into the lipid bilayer as toxic oligomers, which can therein undergo a one-electron oxidation of methionine, resulting in the formation of sulfuranyl radical cations that remove allylic hydrogen atoms from phospholipid acyl chains found in the lipid bilayer, initiating catalytic chain reaction of lipid peroxidation [3,78,84]. Other proposed mechanisms of Met-35 neurotoxicity involve oxidative transfer from methionine to redox metal ions such as copper (II) to form Cu^+^, with resultant onset of Fenton chemistry, i.e., reaction of Cu^+^ with the relatively high concentration of non-polar H_2_O_2_, which would generate hydroxide anions and highly reactive hydroxyl free radicals to initiate lipid peroxidation that would result in a significant level of the neurotoxic product HNE [3,85,86,87,88].

A number of studies showed that when the rate of fibril formation with Aβ(1-42) was lowered, protofibrils were not formed when Met-35 is oxidized to methionine sulfoxide [89,90,91]. Postmortem studies also showed the presence of high levels of oxidized Met-35 in Aβ-rich senile plaques [92,93] and were proposed to be related to the reduced activity of methionine sulfoxide reductase [94], an enzyme that cycles oxidized Met back to its reduced form in AD brains. In addition, studies centered around familial AD and individuals with Down syndrome, regarding trisomy 21, who develop AD-like neuropathology and dementia at ages earlier than sporadic AD patients, further provided a strong association of the role Aβ (*APP* is localized to chromosome 21) in AD pathogenesis and progression [95,96,97,98].

## 4. Evidence of Oxidative Stress in Brain of Subjects with AD and MCI

### Protein Carbonyls in AD and MCI Brains

Protein carbonyl levels were reported to be elevated in MCI and AD brains [99,100,101,102,103]. Smith et al. (1991) showed increased protein carbonyl levels in neurofibrillary tangles, neuronal cell bodies, and apical dendrites, as well as neuronal and glial nuclei in hippocampal sections of AD brains [10]. Further, increased levels of protein carbonyls, diene conjugates, and lipid peroxides were found in the frontal cortex of a Swedish APP670/671 FAD mutation AD model [104]. Studies from our laboratory showed a 42% and 37% increased protein carbonyl content in the hippocampus and inferior parietal lobule of Alzheimer’s disease brains, respectively, relative to AD cerebellum, whereas control brains showed relatively small amounts of similar levels of carbonyl content in these three brain regions [66,101,102,105,106,107,108,109,110]. This increase in protein carbonyl levels is correlated with increased levels of carbonyl reductase (CR), which is increased in brains of individuals with AD and Down syndrome [111], suggesting that the levels of this enzyme might be increased in response to elevated protein carbonyls. Though the level of CR is increased, its functionality remains to be tested. We speculate that this enzyme being oxidatively damaged and not cleared by protein degradation pathways leads to its increased levels in AD brains relative to controls. Elevated oxidative stress was reported to inhibit the function of the 26S proteasome subunit in AD [112,113]; it is possible that the 20S proteasome function might be compromised as well by elevated oxidative stress in AD.

A previous study showed that the levels of protein carbonyls were significantly increased in synaptic and non-synaptic mitochondria in the frontal cortex of AD, consistent with the notion that these elevated protein carbonyl levels would lead to altered mitochondrial function and subsequent increased oxidative stress [114]. Increased levels of protein carbonyls were reported in mitochondria isolated from AD lymphocytes [109]. The increased oxidative stress markers and decreased antioxidant system were also reported in MCI brains [112,113], supporting the concept that oxidative stress plays a significant role in the pathogenesis and progression of AD.

Several oxidatively modified proteins have been detected in AD and MCI brains by using redox proteomics. Butterfield’s research group was the first to utilize redox proteomics to identify specific targets of carbonylation in AD brains [101,102,115]. After this study, several other targets of oxidation were reported from our laboratory in different brain regions, and we also showed the alteration of oxidatively modified protein functions [116].

## 5. Carbonylated Proteins in Brains of Subjects with AD and MCI

Many proteins were found to be modified by protein carbonylation in AD and MCI brains, which supports the observation of altered energy metabolism, structural changes, tau hyperphosphorylation, altered Aβ production, mitochondrial dysfunction, pH alterations, etc. Protein oxidative modification (i.e., nitration, carbonylation, the subjects of this current review) almost always alters protein functionality [109,116].

### 5.1. Carbonylated Proteins in Brains of Subjects with MCI

MCI brains demonstrated increased levels of protein carbonyls [3,106,116]. Redox proteomics revealed an increased protein carbonylation of carbonic anhydrase II (CA II), heat shock protein 70 (Hsp70), mitogen-activated protein kinase I (MAPKI), syntaxin-binding protein I (SBP1), alpha-enolase, GS, pyruvate kinase M2 (PKM2), and Pin1 in MCI brains compared to age-matched controls.

One of the targets identified as an oxidatively modified protein in MCI is Hsp70. Hsp70 is a neuroprotective protein, assisting nearby produced proteins from the ribosomes to fold properly. Altered function of this protein could lead to increased numbers of damaged proteins in the neurons, more than the capacity of the proteasomal system can clear, resulting in the deposition of protein aggregates in MCI brains [106]. This protein is also found to be oxidatively modified in AD brains. Additionally, several other heat shock proteins have been found to be oxidatively modified in AD, including Hsp90 and Hsp60, while Hsp 27 and Hsp 32 are elevated in amnestic MCI [102,108,117]. Another target of protein oxidation is pyruvate kinase in MCI brains [113]. This enzyme is involved in the final step of the glycolysis pathway, important in glucose metabolism and the subsequent regulation of cellular energetics. Pyruvate kinase catalyzes the conversion of phosphoenolpyruvate to pyruvate with the concurrent transfer of the phosphate group from phosphoenolpyruvate to ADP, thereby generating ATP. Pyruvate under aerobic conditions is transported to the mitochondria, where it is converted to acetyl coenzyme A; entering the TCA cycle, the latter is completely metabolized to water and carbon dioxide and during this process more ATP is produced through oxidative phosphorylation by the electron transfer chain and ATP synthase. The observation of decreased brain energetics in MCI and AD brains as observed by PET are likely related to the oxidative modification of proteins involved in glucose metabolism [4].

Another protein that is found to be oxidatively modified is syntaxin-binding protein I (SBP1), a neuron-specific protein that binds strongly to syntaxin 1. Syntaxin is important for synaptic vesicle exocytosis and neurotransmitter release, a key process for neurotransmission. Oxidation of syntaxin-binding protein 1 could impair neurotransmission and consequently might contribute to a loss of synaptic neurotransmission and neuronal function in AD and MCI. Moreover, oxidatively modified SBP1 could be a player in the loss of memory and cognition associated with neurodegenerative processes involved in the progression of MCI to AD. Other proteins that were identified as oxidatively modified are mitogen-activated protein kinases (MAPKs) that are indicated as a possible key regulator in the formation of SP and NFT. In early AD brains, ERK, an MAPK, activation was reported in astroglia, while in more advanced AD, it is associated with neuronal cell bodies and dystrophic neurites around plaques, suggesting that ERK activation in astroglia may be an important early response to the onset of AD pathology [118]. In postmortem AD brains, increased activity of ERK1/2 corelated with abnormal phosphorylation of tau, suggesting a possible role of MAPKs in the hyperphosphorylation of the tau protein [118].

Interestingly, Pin1 also was identified as excessively carbonylated and a dysfunctional protein in MCI [111,114,116,119]. This protein plays an important role in regulating the phosphorylation–dephosphorylation of APP, tau protein, and other proteins like cyclin-dependent kinase-5, etc., which are all important in AD [120,121,122,123]. Oxidatively dysfunctional Pin1 favors the formation of NFTs and senile plaques and traps neurons in the cell cycle, which leads to apoptotic death [124].

### 5.2. Carbonylated Proteins in Brain of Subjects with AD

Redox proteomics led to the identification of specific targets of protein oxidation in AD brains [124,125,126,127,128,129,130] which include, among others, phosphoglycerate mutase (PGM1), glutamine synthase (GS), triosephosphate isomerase (TPI), heat shock cognate 71, creatine kinase BB (CK), alpha enolase (Eno1), peptidyl prolyl cis-trans isomerase 1 (Pin1), ubiquitin carboxyl terminal hydrolase L-1 (UCHL-1), dihydropyrimidinase-related protein 2 (DRP2, also designated CRMP2), ATP synthase alpha chain (α-ATP synthase), gamma- SNAP, and carbonic anhydrase 2 (CA2) [124,125,126,127,128,129,130]. The proteins that were detected might be more susceptible to oxidative damage. With advances in technology, more and more targets of oxidative damage can be identified. Redox proteomics identified those proteins consistent with AD histopathological hallmarks, such as altered neuronal communication, decreased energetics, etc. Interestingly, some proteins were observed as common targets of oxidative modification such as α-enolase, indicating that such proteins might be important in the transition and progression of AD. These proteins are grouped together based on the functional role and their relationship to the state of AD (see Table 1 below).

#### 5.2.1. Carbonylated Proteins Lead to Decreased Glucose Metabolism in AD and MCI

The brain uses glucose as a primary source of energy, utilizing 20% of dietary glucose. PET images from AD brains show marked decreases in glucose metabolism [131] which could be related to the identification of proteins such as CK, ENO1, TPI, PGM1, and α-ATP synthase as carbonylated proteins. Increased carbonylation of these proteins in almost all examined cases is associated with dysfunction, supporting the observed decrease in glucose utilization in different areas in PET scans of the brain in AD patients [131]. Decreased brain energetics start at different MCI levels and progress rapidly in AD. Carbonylation of these proteins involved in glucose metabolism supports the hypothesis that altered energy metabolism is a common mechanism in neurodegenerative diseases, not just AD. ATP is extremely important at nerve terminals for normal neurotransmission. Decreased levels of cellular ATP may lead to altered synaptic transmission and eventually to loss of memory due to altered cellular function or cell death, which starts in early stages, including MCI. Similarly, decreased ATP levels could affect ion-motive ATPase function and thereof affect voltage-gated Ca^2+^ channels, leading to excess Ca^2+^ entry across the neuronal plasma membrane and consequent neuronal death.

Alpha enolase catalyzes the conversion of 2-phosphoglycerate to phosphoenolpyruvate in the glycolytic pathway. This enzyme demonstrated increased oxidation in AD and Aβ models of AD, suggesting that Aβ plays an important role in the oxidation of proteins [102,124]. Further, the activity of the Alpha enolase enzyme was reported to be reduced in MCI and AD brains. Enolase is not just involved in energy metabolism; it is also known to play a role in plasminogen regulation through the MEK/ERK pathway. Plasminogen undergoes proteolysis by tissue-type plasminogen activator (TPA) and is converted to its active form, plasmin. TPA is brain specific, and plasmin in the brain is reported to enhance the degradation of amyloid beta-peptide; loss of function of TPA due to oxidative modification can be correlated with the reported increase in senile plaques in AD. The oxidative modification of alpha enolase may not just disrupt neuronal energy metabolism and ion homeostasis but it can also induce hypothermia, causing abnormal tau phosphorylation through differential inhibition of kinases and phosphatases; it can also enhance the formation of SPs.

CK BB catalyzes the conversion of creatine to phosphocreatine at the expense of ATP, which is later used in the production of high-energy phosphate for the generation of ATP. This protein is reported to be carbonylated in the inferior parietal region of AD compared to age-matched controls using redox proteomics, the activity of CK is diminished in AD brains [101], and loss of its function due to oxidation leads to decreased energetics in neurons and synaptic elements and consequently to impaired brain function in AD. Another, protein that is important in ATP production is ATP synthase, which was identified as excessively carbonylated. This protein undergoes a sequence of coordinated conformational changes of its α and β subunits to produce ATP. The ATP synthase δ subunit is located on the exterior column of the enzyme and is one component of the F_0_ subunit of ATP synthase. With a sufficient proton gradient, the rotor of this mitochondrial complex moves so that ADP and P_i_ bind in a tight conformation and produce ATP. The rotor then moves 120° counterclockwise to the open position, thereby releasing ATP into the cell. ATP synthase has been previously shown to be carbonylated in late-stage AD [124]. The oxidation of ATP synthase leads to the inactivation of this mitochondrial complex and leads to decreased activity of the entire electron transport chain (ETC) and impaired ATP production, resulting in possible electron leakage and increased ROS production, suggesting an alternate rationale for the generation of oxidative stress seen in AD [124].

In AD brains, studies showed an altered expression of mitochondrial proteins, deficiency of functional abilities, and reduced activity in different complexes of the ETC, which might induce changes in complex I, III, and IV, leading to increased electron leakage from the mitochondria to produce ROS. This action may also affect the proton gradient and overall mitochondrial function, which suggests that it is an alternate or complementary mechanism for the generation of oxidative stress in AD [124].

Another enzyme that comes from the glycolytic pathway, the phosphoglycerate mutase that catalyzes the interconversion of 3-phosphoglycerate to 2-phosphoglycerate, was reported to have decreased expression and increased carbonylation in AD brains [124]. This finding is consistent with the decreased glucose metabolism in AD brains, as indicated by PET scans. Interestingly, triose phosphate isomerase (TPI), a glycolytic enzyme that catalyzes isomerization of dihydroxyacetone phosphate to glyceraldehyde-3-phosphate, was also found to be carbonylated, with no change in its activity. This could be attributed to the site of carbonylation not being close to the catalytic area of the enzyme or to the characterization of TPI as “the perfect enzyme” [124].

Taken together, oxidative inactivation of proteins involved in glucose metabolism may explain the known metabolic defects in AD detected by PET scanning.

#### 5.2.2. Carbonylated Protein Causes Loss of Brain Cells in AD

Excessive levels and extended stay of the excitatory neurotransmitter glutamate in the synaptic cleft leads to excitotoxicity. Excess glutamate is normally taken up from the extracellular space via the glutamate transporter, with astrocytes, including EAAT2 (aka Glt-1), present. The glutamate is converted to glutamine by the glutamine synthetase (GS) enzyme, which is important for maintaining the glutamate–glutamine cycle, thereby playing an important role in synaptic transmission and in the regulation of ammonia levels in the brain. Oxidative modification of GS leads to reduced activity which could lead to impairment of the glutamate–glutamine cycle in AD brains [8,124]. Increased glutamate levels lead to respective activation of post-synaptic glutamate receptors, subsequently leading to increased intracellular calcium levels, eventually causing increased activation of calcium-dependent proteins and leading to cell death due to excitotoxicity of neurons. Further, as noted, decreased GS activity affects the neuronal pH due to the accumulation of ammonia, which suggests another possible mechanism for neuronal toxicity.

#### 5.2.3. Carbonylated Protein Causes Accumulation of Protein Aggregates in AD

The ubiquitin–proteasome system is important for the degradation of damaged or misfolded proteins. In AD brains and other neurodegenerative diseases, UCH-L1, which belongs to a family of ubiquitin carboxyl-terminal hydrolases, and accounts for 2% of brain proteins, was found to be oxidatively modified [101,126]. In AD brains, the activity of 26S proteosome activity was reported to be decreased, to which oxidative dysfunction of UCH-L1 may contribute [126]. We hypothesize that oxidative modification and subsequent loss of activity of UCH-L1 in AD brains contributes to the increased accumulation of ubiquitinylated proteins, decreased proteasome activity, and accumulation of damaged proteins in AD brains. Thus, oxidative inactivation of UCH-L1 is consistent with both the protein aggregation and oxidative stress observed in AD brains.

#### 5.2.4. Carbonylated Protein Causes Changes in Synapses

One of the proteins that is reported as an oxidatively modified protein in AD brains is dihydropyriminidase-related protein 2, also known as collapsin response mediator protein 2 (CRMP-2). This protein is critical to neuroplasticity and is important in the process of memory consolidation. DRP-2 plays an important role in regulating the dendritic length via interaction with collapsin. It also helps with the maintenance of microtubule assembly, cellular migration, and cytoskeletal remodeling. In AD brains, the dendritic lengths have been reported to be short [127]. This likely would lead to less efficient neuronal communication with adjacent neurons and subsequently impacting the memory and cognitive decline observed in AD. Further, DRP-2 has been reported to be associated with neurofibrillary tangles, which may lead to decreased levels of cytosolic DRP-2 [128,129]. This protein was also reported to have reduced expression in fetal and adult Down syndrome [128] patients, which further suggests that Aβ might be a key player. As mentioned earlier, one of the clinical diagnostic features of Alzheimer’s disease is a loss of memory, consistent with a loss of DRP2 function and its reduced levels. Synaptic remodeling is an important part of learning and memory, and oxidation of CRMP2 could be linked to alterations in the structure and function of this protein, which, conceivably, is likely involved in the observed cognitive impairments in MCI and AD [102,124].

#### 5.2.5. The Carbonylated Protein Pin1 Is Correlated with Changes in Synapse APP Regulation, Tau Hyperphosphorylation, and Cell-Cycle Regulation in AD and MCI

In the AD and MCI brains, peptidyl-prolyl *cis/trans* isomerase (Pin1) was found to be a common target of oxidation [114,121,130]. Pin1 is a regulatory protein that recognizes phosphorylated Ser-Pro or phosphorylated Thr-Pro motifs in target proteins and causes cis/trans isomerization of the proline amino acid of the substrate, thereby regulating the activity of the target proteins. Both APP and Tau proteins were reported to the substrates of Pin1 [132,133]. Pin1 plays an important role in regulating the phosphorylation–dephosphorylation of APP, tau protein, and other proteins like cyclin-dependent kinase-5, etc. In AD brains, Pin1 levels were found to be reduced and also oxidatively modified [131]. Pin1 was also found to be colocalized with phosphorylated tau and showed an inverse relationship to the expression of tau in AD brains [134,135]. The impaired activity of Pin1, which normally regulates protein phosphatase 2A and GSK-3β, favors the hyperphosphorylation of tau proteins, promoting the formation of NFT. Elevated phosphorylated tau would lead to destabilization of microtubules, which would decrease axonal transport and disassembly of the axonal cytoskeleton. A study conducted by Lu et al. (2000) showed that overexpression of Pin1 could restore the function of tau proteins in an AD model enter the apoptosis process [120]. Neuronal cells are non-dividing cells, unlike other cells in the body; if a post mitotic neuron enters the cell cycle, neurons would enter the apoptosis process. Hence, oxidative modification of Pin1 conceivably could be related to increased levels of cell-cycle proteins in AD and MCI brains [120,136]. This single protein oxidation can be linked to the formation of NFT, SP, and cell death, which are hallmarks of AD [121].

## 6. Protein Nitration in MCI and AD Brain

Levels of nNOS are constitutively expressed in neurons, and in AD these levels are increased. Moreover, levels of iNOS have been reported to be increased in AD brains due to enhanced inflammation [137,138,139,140]. iNOS catalyzes the generation of NO, which has been implicated in the impairment of mitochondrial respiration, synaptic failure, and neuronal cell death during neurodegeneration [17]. One of the signatures of nitrosative stress is tyrosine nitration, a posttranslational protein modification, resulting in the formation of 3-nitrotyrosine residues (3-NT), which can promote structural changes leading to aggregation of proteins [14]. Increased levels of 3-NT in MCI (hippocampus and inferior parietal lobule, IPL) and AD brains have been reported [110,141,142,143], which correlated with the increased expression of iNOS. Further, the Aβ protein is reported to disrupt NO activity, promoting synapses and LTP alterations in AD, and studies of AD have revealed that nitrosative injury has a role in the pathogenesis of AD and the structural and functional changes reported in AD [116,144,145,146,147,148].

### 6.1. Nitrated Proteins in Brains of Subjects with MCI and AD

A study by Kummer et al. (2011) showed that the nitration of Tyr at the 10 position accelerated aggregation of Aβ and the nitrated Aβ was detected in the core of Aβ plaques of APP/PS1 mice and AD brains [149]. Further, these authors also showed that reducing the levels of iNOS with the iNOS inhibitor L-NIL strongly decreased 3NTyr^10^-Aβ and overall Aβ deposition and cognitive dysfunction in APP/PS1 mice. Moreover, the researchers also demonstrated that the injection of 3NTyr^10^-Aβ into the brain of young APP/PS1 mice induced β-amyloidosis, suggesting that modulation of iNOS activity could be a potential therapeutic option to treat AD [149]. Consistent with the nitration of Aβ, Reyes et al. (2008) demonstrated nitration of Tyr 18, followed by Tyr 29 of tau, which is mostly associated with or near amyloid plaques [150]. Redox proteomics studies from the Butterfield laboratory led to the identification of a large number of nitrated proteins in AD brains compared to control brains, including alpha- and gamma-enolase, lactate dehydrogenase (LDH), neuropolypeptide h3, TPI and alpha-actin in AD inferior parietal lobule (IPL) [116], and alpha-enolase, glyceraldehyde-3-phosphate dehydrogenase (GAPDH), ATP synthase alpha-chain, carbonic anhydrase-II, and voltage-dependent anion channel (VDAC) protein in the AD hippocampus [124]. Nitrosylated proteins, DRP2, alpha-internexin, glutamate dehydrogenase 1, alpha-enolase, GFAP, MDH, ProSAAS precursor protein, proopiomelanocortin, proenkephalin, and septin are modified by S-nitrosyl-cysteine modification in the entorhinal cortex of AD in response to Aβ activation of glial cells surrounding SP, which conceivably leads to increased nitrosylation of GFAP, thereby contributing to the pathogenesis of AD. Additionally, protein disulfide isomerase (PDI), an enzyme that catalyzes thiol–disulphide exchange, has been reported to be S-nitrosylated in AD brains, which might lead to alterations in its ability to facilitate disulfide bond formation and rearrangement reactions, consequently leading to increased accumulation of polyubiquitinylated proteins and activation of the ER-resident unfolded protein response (UPR) [151]. Lipton and colleagues also reported increased levels of S-nitrosylation of dynamin-related protein 1 in brains of subjects with AD and suggested that S-nitrosylation of this protein may trigger mitochondrial fission, consequently adding to known mitochondrial damage in AD, which could contribute to synapse loss and neuronal damage in this disorder [143,152]. Further, the Butterfield group also identified α-enolase, glucose-regulated protein precursor (GRP), aldolase, glutathione-S-transferase Mu (GST M), multidrug-resistant protein-3 (MRP3), 14-3-3 protein gamma, MDH, peroxiredoxin 6 (PR VI), DRP-2, fascin 1, and heat shock protein A8 (HSPA8) as specifically 3-NT-modified proteins in MCI brains compared to age-matched controls [110]. These nitrated proteins are involved in various cellular functions such as energy metabolism, structural maintenance, pH regulation, and mitochondrial function. There are some overlapping targets of protein nitration between AD and MCI, suggesting their important role in the progression of this devastating disorder.

#### 6.1.1. Nitrated Protein Likely Causes Altered Cellular Energetics in AD

Enolase, GAPDH, TPI, and LDH are proteins of the glycolytic pathway. And MDH, ATP synthase alpha-chain, and voltage-dependent anion channel (VDAC) proteins are involved in cellular energetics via the Krebs cycle or the ETC. α-Enolase is reported to be the target of both protein nitration and carbonylation in MCI and AD brains, contributing to the observation of decreased glucose metabolism in AD and MCI brains [4,110,116]. As mentioned earlier, this protein might be important in buildup of Aβ, leading to senile plaque formation and also altered cellular signaling and activation of cell survival pathways [153]. Interestingly, nitrated TPI in AD brains (hippocampus and frontal cortex) did not show any change in its function [124,154], suggesting that the site of modification and amino acid composition of the protein determines if the oxidative modification has an effect in inducing the change in the folding pattern and the structure of the protein. GAPDH is a key enzyme in the glycolytic pathway. Dysregulation of the multiple functions of this protein is found in AD and MCI brains [155]. For example, GAPDH dysfunction results in increased levels of upstream glycolytic intermediates such as glyceraldehyde-3-phosphate, which can activate the glycation pathway—leading to the formation of methylglyoxal—which can react with biomolecules, causing further damage and altered cellular function [156]. Additionally, GAPDH nitration can lead to increased activation of the protein kinase C pathway and decreased levels of GSH via the polyol pathway [157]. GAPDH has been reported to regulate cellular transcription, cellular signaling, and vesicular transportation, in addition to binding to other small molecules such as nitric oxide (NO), glutathione (GSH), tumor necrosis factor (TNF)-α, etc. [155]. GAPDH also interacts with β-amyloid precursor protein (AβPP) [158]. Longitudinal studies have shown a relationship between AD and glucose dysmetabolism [4,159]. Investigations showed that diabetic patients are also at an increased risk of developing AD, suggesting strong links between altered glucose dysmetabolism, insulin, and AD [4,160]. Specifically, an enzyme that regulates the levels of insulin in AD, i.e., insulin-degrading enzyme (IDE), is affected in AD [161]. This enzyme is also reported to be important in the degradation or removal of Aβ from cells [162]. Altered levels of IDE are linked to increased levels of Aβ accumulation and are proposed to play an important role in SP [161]. In addition to decreased IDE levels, decreased activity of the enzymes involved in glucose metabolism by oxidation leads to increased glucose accumulation [4].

The reduced levels of insulin appear to induce GSK-3β activity, which might lead to increased phosphorylation of tau protein and consequently NFT formations [163]. Increased glucose levels can also lead to increased advanced glycation end product (AGE) formation, which have toxic effects on neurons through osmotic insults and oxidative stress. The increased levels of AGEs can also activate microglia in the CNS, further promoting formation of free radicals and enhanced inflammatory markers [164]. A number of studies also showed that microglia are one of the underlying mechanisms of AD pathogenesis. Normal levels of glucose are important in meeting cellular ATP to regulate the functions of neuronal cells by regulating various pathways and cognition. A study showed that insulin protects neurons against β-amyloid-derived diffusible ligand (ADDL)-induced synaptic loss and induced oxidative stress [164]. Interestingly, studies showed that insulin protects neurons not by simply binding to ADDL but rather via an insulin-dependent signaling mechanism. Insulin reportedly regulated the expression of *N*-methyl-D-aspartate (NMDA) receptors, one of the calcium (Ca^2+^)-regulating proteins which regulates the functions of other proteins that are important in the learning and memory process, a process altered in AD brains. Further, acetylcholine transferase, an enzyme involved in the synthesis of acetylcholine, was reported to be influenced by insulin [165]. Memory and attention in AD patients improved after intranasal insulin administration, a method that avoids effects of systemic glucose levels, thereby causing disruption of systemic insulin-related glucose metabolism [166].

#### 6.1.2. Nitrated Protein Causes Altered Cell Cycle and Pathological Hallmarks of AD

As discussed earlier, Pin1 was identified as a carbonylated protein in AD and MCI brains with decreased levels [121,167]. Pin1 plays an important role in regulating crucial regulatory functions for intracellular processes. As stated above, this protein is reported to directly and indirectly regulate APP processing and tau phosphorylation, resulting in SP and NFT, respectively. The non-amyloidogenic processing of APP was reported to be regulated by Pin1, acting on the intracellular C-terminal domain (AICD) pT668-P of APP [168]. Further, Pin1 was reported to interact with and inhibit the kinase activity of GSK3β, an enzyme involved in the phosphorylation of the tau protein, promoting hyperphosphorylation and also promoting APP phosphorylation, which favors the amyloidogenic processing of APP [169]. Pin1 has been shown to induce dephosphorylation of Tau at specific sites via regulation of protein phosphatase 2-A (PP2A) and the knock-down of Pin1 leads to hyperphosphorylation and aggregation of Tau-positive NFTs [169]. Pin1 also is known to play an important role in regulating the cell cycle, and in AD a large number of control proteins important to the cell cycle have been identified as having aberrant expression, localization, or post translational modifications [170,171]. Further, the levels of a number of proteins involved in cell-cycle regulation such as CDK2, CDK5, and cyclin G1 were reported to be elevated in both the hippocampus and IPL in brains of subjects with aMCI, demonstrating that cell-cycle changes may appear prior to classical AD dementia [121,170,171,172,173].

#### 6.1.3. Nitrated Proteins Cause Altered Neuronal Structure, Function, and Signaling

The 14-3-3-protein gamma protein is involved in several cellular functions including signal transduction, protein trafficking, and metabolism. This protein was reported to be one of the nitrated proteins in MCI IPL. Elevated levels of this protein were reported in the AD brain [174], CSF [175], and the ICV animal model of AD [176], which conceivably could lead to altered binding to two of its normal binding partners, i.e., glycogen synthase kinase-3β (GSK3β) and tau, and may promote tau phosphorylation and polymerization, potentially to the formation of NFT. Another protein, fascin 1 (FSCN1), also known as p55, is a structural protein involved in cell adhesion and cell motility [177,178] and is used a marker for dendritic function. FSCN1 also interacts with protein kinase C (PKC) [179], thereby playing an important role in post-translational protein modification, and intracellular signaling protects cells from oxidative stress. FSCN1 and modification of the protein is consistent with increased oxidative and nitrosative stress in AD and MCI brains and consistent with impairment of synaptic transmission, contributing to the progression of AD. Further, biliverdin reductase (BVR), a dual-specificity Ser/Thr and Tyr kinase, interacts with and modulates the function of the insulin growth factor-1 (IGF-1) and MAPK pathways and regulates the expression of various oxidative stress adaptive responsive genes [180,181]. The BVR-A protein showed increased expression and nitration in both AD and MCI brains, suggesting alterations of several important metabolic pathways that are regulated by BVR-A, including upstream insulin signaling [182,183].

## 7. Summary and Conclusions

Table 1 summarizes the functional classes of carbonyl- or 3-nitrotyrosine-modified proteins from the hippocampus or inferior parietal lobule brain regions as a function of the stage of the progression of AD, compared to those same brain regions from aged-matched normal control individuals. All autopsied brain specimens were obtained at short post-mortem intervals (less than approximately 4 h). In the opinion of the authors of this paper, short PMIs are necessary to make meaningful conclusions regarding oxidative damage to brains in AD and MCI and in corresponding controls. Oxidative modification of proteins both in the MCI and AD brain suggests that they play an important role in the pathophysiology and progression of AD [3,4,184]. The appearance of common targets of oxidation of proteins between MCI and AD further implies the important roles of oxidative and nitrosative stress in contributing to Aβ and tau pathology, mitochondrial dysfunction, synaptic impairment, and neuroinflammation. Clinical signs and symptoms of MCI and AD are certainly correlated with oxidative damage and conceivably could be caused in part by oxidative damage.

Currently available strategies to mitigate protein oxidation in aging brains include antioxidant therapies, lifestyle interventions, and targeted therapies [3]. Antioxidants such as vitamin E, vitamin C, and coenzyme Q_10_ have been explored for their potential to mitigate protein oxidation and oxidative stress in the brain. Natural bioactive molecules, often incorporated into the diet, can become part of a widely adopted approach to attempt to avoid or at least delay the onset of dementia in AD. Moreover, the treatment of patients with approved drugs to delay the progression of AD is a therapeutic strategy worthy of exploration. Clinical antioxidant trials have shown mixed results [3]. Some compounds were effective in animal models but did not prove effective in human clinical trials. Further, regular physical exercise, a balanced diet rich in antioxidants, and adequate sleep are lifestyle factors that can help reduce oxidative stress and support brain health during aging [185]. These interventions can enhance the brain’s natural defense mechanisms against protein oxidation. Additionally, developing targeted therapies that specifically address protein oxidation in the brain represents a promising approach for combating age-related neurodegenerative disorders. These therapies may include small molecules that can prevent protein oxidation or enhance the clearance of damaged proteins. We posit that continued investigation into the role of oxidative damage in the pathogenesis and progression of AD will shed more insights into this important mechanism of neuronal loss and cognitive decline in this devasting disorder.

## Figures and Tables

**Table 1:** Carbonylated- or 3-nitrotyrosine-modified hippocampal or inferior parietal lobule proteins in MCI or AD Brains with consequent diminution of function * Identified by redox proteomics in the butterfield laboratory.

Functional Class	Stage of AD **	Protein
Glucose Metabolism		
	MCI	Glucose-regulated Protein Precursor
	MCI; EAD	Aldoase
	LAD	Triose Phosphate Isomerase
	LAD	Glyceraldehyde-3-Phosphate Dehydrogenase
	EAD; LAD	Phosphoglycerate Mutase 1
	MCI; EAD; LAD	a-Enolase
	MCI	Pyruvate Kinase M2
	MCI	Lactate Dehydrogenase
	LAD	Creatine Kinase BB
	LAD	Aconitase
	MCI; EAD; LAD	ATP-Synthase
Cellular Redox Homeostasis		
	MCI	Glutathione-S-Transferase Mu
	MCI	Peroxiredoxin 6
Synaptic Plasticity; Cytoskeletal Protein Structure; Vesicle-mediated Transport; Neurotransmission		
	LAD	Carbonic Anhydrase II
	MCI; LAD	Dihydropyrimidinase-related Protein-2 (also known as Collapsin Response Mediator Protein-2)
	MCI	Fascin 1
	LAD	Neuropolypeptide h3
	MCI	β-Actin
	LADee	α-Tubulin
	MCI	Syntaxin-binding Protein 1
	LAD	γ-Synaptosomal Protein-Like Soluble N-ethylmaleimide-sensitive Factor
	LAD	Voltage-dependent Anion-channel Protein 1
Excitotoxicity		
	MCI; LAD	Glutamine Synthetase
Protein Folding and Degradation		
	LAD	Heat-shock Cognate 71
	MCI	Heat-shock Protein 70
	MCI	Heat-shock Protein 90
	LAD	Ubiquitin Carboxy-terminal Hydrolase L-1
Protein Regulation; Signal Transduction		
	MCI; LAD	Peptidylprolyl-cis-trans Isomerase
	MCI	14-3-3-γ
	MCI	Mitogen-activated Protein Kinase 1
Neuroinflammation		
	EAD	Glial Fibrillary Acidic Protein

* Except that of triose phosphate isomerase; even though this protein was oxidatively modified in late-stage AD brains, its function was not altered from that of control brains, as noted in the text. ** MCI = mild cognitive impairment; EAD = early Alzheimer’s disease; LAD = late Alzheimer’s disease.
